# Optimality and evolution of transcriptionally regulated gene expression

**DOI:** 10.1186/1752-0509-5-128

**Published:** 2011-08-16

**Authors:** Frank J Poelwijk, Philip D Heyning, Marjon GJ de Vos, Daniel J Kiviet, Sander J Tans

**Affiliations:** 1 AMOLF Institute, Science Park 104, 1098 XG, Amsterdam, The Netherlands; 2 Green Center for Systems Biology and Department of Pharmacology, University of Texas Southwestern Medical Center, Dallas, TX 75390-9050, USA

## Abstract

**Background:**

How transcriptionally regulated gene expression evolves under natural selection is an open question. The cost and benefit of gene expression are the driving factors. While the former can be determined by gratuitous induction, the latter is difficult to measure directly.

**Results:**

We addressed this problem by decoupling the regulatory and metabolic function of the *Escherichia coli lac *system, using an inducer that cannot be metabolized and a carbon source that does not induce. Growth rate measurements directly identified the induced expression level that maximizes the metabolism benefits minus the protein production costs, without relying on models. Using these results, we established a controlled mismatch between sensing and metabolism, resulting in sub-optimal transcriptional regulation with the potential to improve by evolution. Next, we tested the evolutionary response by serial transfer. Constant environments showed cells evolving to the predicted expression optimum. Phenotypes with decreased expression emerged several hundred generations later than phenotypes with increased expression, indicating a higher genetic accessibility of the latter. Environments alternating between low and high expression demands resulted in overall rather than differential changes in expression, which is explained by the concave shape of the cross-environmental tradeoff curve that limits the selective advantage of altering the regulatory response.

**Conclusions:**

This work indicates that the decoupling of regulatory and metabolic functions allows one to directly measure the costs and benefits that underlie the natural selection of gene regulation. Regulated gene expression is shown to evolve within several hundreds of generations to optima that are predicted by these costs and benefits. The results provide a step towards a quantitative understanding of the adaptive origins of regulatory systems.

## Background

Evolution is often viewed as an optimization process, in which selection drives the fixation of fitness-increasing mutations until the optimal phenotype that maximizes fitness is reached [1-5]. It is routinely observed that organisms, when challenged with novel environments, can adapt and increase fitness by genetic changes [6-8]. However, adaptation alone does not necessarily imply optimality, as for instance constraint of genetic or physico-chemical origins may prevent access to optimal phenotypes [9,10]. Investigating optimality requires a mechanistic understanding of the studied phenotype and its relation to fitness, which is seldom available. Consequently, it remains a challenge to assess whether observed phenotypes represent optimal solutions to the demands imposed by the environment.

These limitations can be overcome by focusing on a well-understood phenotype and mapping its relation to fitness, as has been shown by a few recent studies. For instance, *Escherichia coli *cells evolved by experimental evolution to predicted optimal metabolic fluxes, using a computer model of the metabolic network [3]. In another study [4], a gene involved in metabolism that was completely induced and hence effectively unregulated was shown to evolve to optimal expression levels, which could be predicted using measurements of the costs involved in protein expression [11,12].

Here we ask a related but different question, namely whether the evolution of transcriptionally regulated gene expression is also predicted by a cost-benefit analysis. In contrast with constitutive gene expression studied previously [4], the level of expression in our case depends strongly on the binding of a transcription factor to the regulatory region, in addition to the more generic parameters such as the strength of the promoter and the ribosome binding site. Understanding how this regulatory control mechanism affects the dynamics of evolution by natural selection is the central motivation of this study.

**Figure 1:**
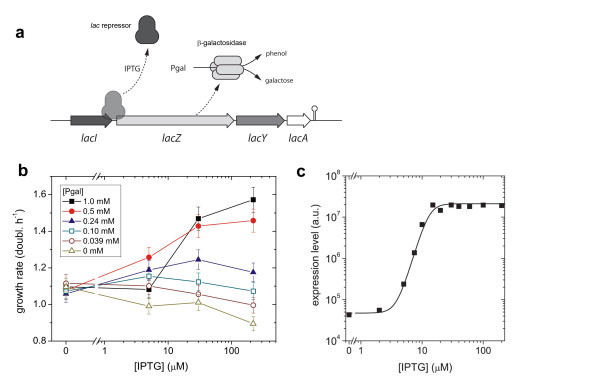
**Experimental design and characterization**. a) Schematic diagram of the *lac *operon illustrating the decoupling of regulation and metabolism using IPTG that induces but is not metabolized, and Pgal that is metabolized but does not induce. b) Measured growth rates *g(I, P) *for a number of combinations of IPTG and Pgal in a minimal M9 medium plus casamino acids (see Materials and Methods). At low Pgal concentrations, inducing the *lac *operon products results in an expression cost that is larger than the benefit, resulting in a decrease of the growth rate. For higher Pgal concentrations the benefit will dominate the cost. Each data point is obtained from three separate measurements. c) The wild-type induction profile $Z_{\text{reg}}^{\text{WT}}\left(I\right)$ in the absence of Pgal, fitted with a Hill curve (Additional file 1).

The *E. coli *lactose operon is well suited for such an investigation, given the wealth of available functional and mutational data [13]. Despite these advantages however, some experimental challenges remain. To predict the balance between costs and benefits of expression, both must be measured for a range of *lac *operon expression levels, but at constant lactose concentrations. The latter is not possible with the wild-type *lac *operon, as its expression level explicitly depends on the lactose concentration. Here we achieve a decoupling of the metabolic and inductive properties of the environment, using the compounds phenyl-β-D-galactoside (Pgal), which can be metabolized but does not induce, and isopropyl-β-D-thiogalactopyranoside (IPTG), which cannot be metabolized but does induce (Figure 1a). This approach allows us to measure both the cost and benefit of *lac *operon expression for independently varying levels of inducer and carbon source, and to directly determine the optimum expression level of the metabolic genes for each carbon source concentration. Moreover, the decoupling allows one to study the evolution of regulated expression in a controlled way; by establishing environments in which the wild-type *lac *regulation is suboptimal (the level of gene expression set by induction does not match the optimum expression level demanded by the amount of carbon source in the environment.) We generated several constant and alternating environments where the optimum regulatory responses differ from wild-type, and used an experimental evolution approach [7] to explore the adaptation of *lac *regulation to these new conditions.

## Results and discussion

### Optimality in regulated gene expression

Following a recent study [4], we describe the growth rate of a population of *E. coli *cells as a function of expression of metabolic genes and carbon source in the environment in terms of the cost and benefit of gene expression

(1) $$g=g_{0}-\eta \left(Z\right)+B\left(Z,L\right)$$

where *g_0 _*is the basal growth rate, set by compounds other than lactose in the environment. *η(Z) *is the decrease of growth rate due to the metabolic burden of producing *lac *operon gene products LacZ, LacY, and LacA [11,12]. *B(Z, L) *is the growth advantage due to lactose metabolism, which depends on both the expression level, *Z*, of the *lac *gene products (in particular LacZ), and the concentration of lactose in the environment *L*. As both *η(Z) *and *B(Z, L) *are increasing functions of the expression level *Z*, equation (1) predicts that for each concentration of lactose in the medium there will be an optimum expression level *Z = Z*_opt_(*L*) where benefit minus cost is maximal. Which expression level is optimal depends on the properties of the regulated proteins, such as their Michaelis-Menten kinetics and transport properties.

As we focus here on the adaptation of the regulated *lac *operon expression, we explicitly incorporate the dependency of the expression level on the lactose concentration by substituting *Z = Z*_reg_(*L*), which yields

(2) $$g=g_{0}-\eta \left(Z_{\text{reg}}\left(L\right)\right)+B\left(Z_{\text{reg}}\left(L\right),L\right)$$

where *Z*_reg _describes the system's regulatory properties and is referred to as the regulatory response or induction profile. Now a gene regulatory system can be said to be optimally adapted if it satisfies

(3) $$Z_{\text{reg}}\left(L\right)=Z_{\text{opt}}\left(L\right)$$

which implies that the regulatory system establishes a connection between the inductive properties and the catabolic payoff of lactose. At low levels of lactose, where the cost term will dominate the benefit term, the optimal expression level will be low or zero. Conversely, at high lactose concentrations the optimal expression level will be high. It is important to note that this criterion for regulatory optimality only concerns the relation between expression levels and catabolite concentrations. The regulatory system may also be subject to optimization for response times [14], structural architecture [15], robustness to either mutation [16], protein number fluctuations [17,18], or otherwise.

### Optimality for decoupled *lac *regulation

When organisms are challenged by a new environment, they may perform sub-optimally and undergo selection towards a new phenotypic optimum. One method to establish such directional selection in controlled experiments would be to modify the regulatory response or downstream regulated genes by genetic modifications. Another approach, which we introduce here, is to decouple inducer and carbon source and allow the regulatory system to adapt to a new relation between the two. An additional advantage is that a selective pressure can be applied to the wild-type *lac *operon, and does not require modification of the regulatory system.

For the *lac *system, a large number of artificial compounds have been synthesized [13], that interact with the gene products in a different way than lactose. The decoupling between *lac *signaling and metabolism can be achieved by using isopropyl-β-D-thiogalactopyranoside (IPTG), and phenyl-β-D-galactoside (Pgal). IPTG is a gratuitous inducer; it binds to the *lac *repressor and relieves repression, but cannot be hydrolyzed by β-galactosidase (LacZ). Pgal, on the other hand does not induce LacI, but is hydrolyzed by LacZ, releasing galactose (for further metabolism) and phenol. Now the optimality relation reads

(4) $$Z_{\text{reg}}\left(I\right)=Z_{\text{opt}}\left(P\right)$$

where *I *and *P *(the IPTG and Pgal concentrations in the environment) are independent variables. Relation (4) states that for each Pgal concentration *P *there is an IPTG concentration *I *that achieves an optimum expression level. In the same vein, IPTG concentrations exist that yield suboptimal expression levels. In the latter case, the original regulatory response $Z_{\text{reg}}^{\text{WT}}\left(I\right)$ may evolve to $Z_{\text{reg}}^{\text{MUT}}\left(I\right)$ that does achieve the optimal expression level. Note that *Z*(*P*) may also incur evolutionary changes, which would correspond to β-galactosidase optimizing to Pgal metabolism. With inducer and carbon source decoupled, the equation for the growth rate (2) now reads:

(5) $$g\left(I,P\right)=g_{0}-\eta \left(Z_{\text{reg}}\left(I\right)\right)+B\left(Z_{\text{reg}}\left(I\right),P\right)$$

**Figure 2:**
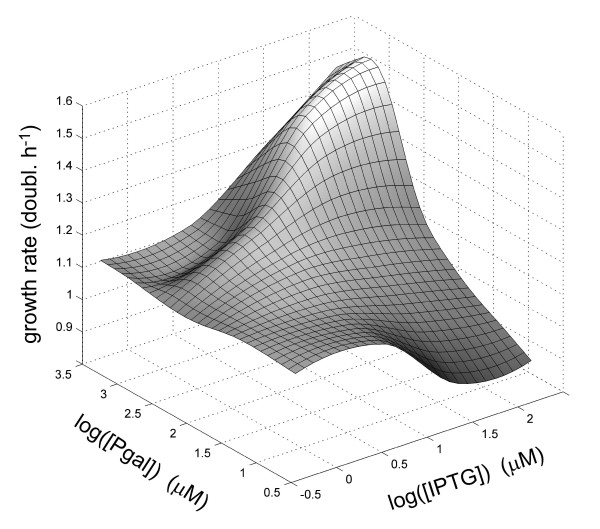
**Growth rate *g(I, P) *as a function of IPTG and Pgal, interpolated and smoothened from Figure 1**. The decoupling of inducer and carbon source is evident: addition of Pgal when not expressing *lac *operon genes (low IPTG concentration) will not result in growth rate increases, and addition of IPTG without Pgal will lower the growth due to an expression cost. The ridge in the landscape is caused by anti-induction of the *lac *repressor by Pgal, when present at a high concentration (see Additional file 1). For low Pgal concentrations we used a functional relation for the cost of expression fitted to the data (Additional file 1).

We determined growth rates *g(I, P) *of *Escherichia coli *MG1655 cells (termed 'wild-type' hereafter) [19] carrying the *lac *operon, as function of IPTG and Pgal (Figure 1b and 2). These two compounds are added to a casamino acids minimal medium, which confers a basal growth rate *g_0 _*of 1.09 generations per hour. A wild-type induction profile $Z_{\text{reg}}^{\text{WT}}\left(I\right)$, measured using the fluorogenic substrate FDG (see Materials and Methods), is shown in Figure 1c. Note that we observed expression decreases at higher concentrations of Pgal, which can be explained by the competitive binding of Pgal to the inducer binding site of the repressor (see Additional file 1). The absence of this anti-induction effect at lower Pgal concentrations is consistent with the known affinities, which is much higher for IPTG than for Pgal (a *K_D _*of 1.10^-6 ^M versus 1.10^-3 ^M [20]). All experiments presented hereafter were performed in this low Pgal regime.

The growth data (Figure 1b and 2) shows that in the absence of Pgal (where basic growth is supported by casamino acids), induction results in a decrease of the growth rate. This suggests that expression of the operon withdraws resources that could otherwise be used for cell growth. At full induction ([IPTG] > 200 μM), this cost to *lac *operon expression results in a reduction in growth rate of about 0.2 doublings per hour. The addition of Pgal has the opposite effect on growth rate. Increasing concentrations of Pgal increase the growth rate, initially compensating for the protein expression costs, and eventually resulting in an overall growth rate increase of up to 1.6 doublings per hour. These growth rate increases indicate a benefit of *lac *operon expression originating from Pgal metabolism.

The total fitness or growth rate, or the expression benefits minus the costs, achieves a maximum value at a certain optimal inducer concentration, as can be seen directly in the measured data (Figure 1b). In the absence of Pgal, it is optimal to have no induction. At 0.10 mM and 0.24 mM Pgal, the growth rate is maximized for IPTG concentrations near 5 μM and 30 μM respectively. For higher Pgal concentrations the maximum observed growth rates are at inducer levels of 200 μM or higher.

**Figure 3:**
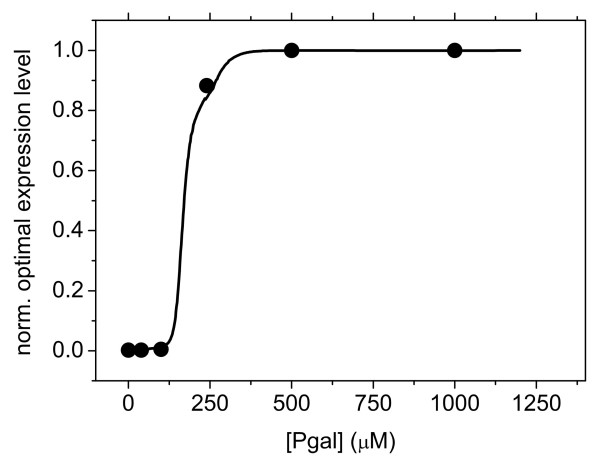
**Optimal expression levels of *lac *operon genes *Z*_opt_(*P*) as a function the Pgal concentration *P *in the environment**. The black curve is obtained from the landscape in Figure 2 and the solid circles represent the optimal expression levels obtained directly from the growth data in Figure 1.

We determined the optimal expression levels, *Z*_opt_(*P*) using the smoothened growth data (Figure 2) and the induction profiles that were separately measured for different concentrations of Pgal (Additional file 1). This optimality relation is given in Figure 3 (black curve), together with the optimal Pgal concentrations (solid circles) as obtained directly from the growth data in Figure 1. Although the optimal expression level shows a sharp Pgal dependence, this does not necessarily imply that the strength of selection on expression is strong. On the contrary; the inflexion point of Figure 3 lies at a Pgal concentration of ~150 μM, and at this Pgal concentration the fitness landscape in fact shows a weak dependence on expression (Figure 2). This suggests that at these Pgal concentrations, suboptimal expression will result only in weak selective pressures.

**Figure 4:**
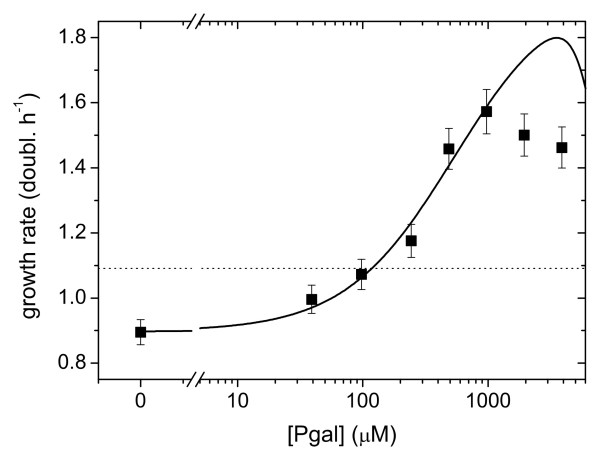
**Fit of growth data at high induction (220 μM IPTG) using a reaction kinetics model (Additional file 1)**. The dotted horizontal line indicates the growth rate in the absence of Pgal and IPTG. The growth difference between this line and the data point at [Pgal] = 0 represents the cost of protein expression. For higher Pgal concentrations this cost is compensated and eventually dominated by the benefit of Pgal metabolism. Cost and benefit are balanced for a Pgal concentration of 1.2∙10^2 ^μM.

The cost and benefit in our system were modeled by equation (5). Because in our system induction and metabolic properties are separated, we adjusted the model to include IPTG induction and anti-induction for high concentrations of Pgal (Additional file 1), using independent measurements of the expression levels of LacZ. At high IPTG concentrations (Figure 4, for 220 μM IPTG), the model and data show a quantitative agreement, with the model accurately predicting that cost and benefit balance at a Pgal concentration of 120 μM. However, the model does not describe the data quantitatively for lower IPTG concentrations up to 5 μM (Additional file 1): this regime shows only a marginal rise in expression levels (Figure 1b), and hence only a marginal increase of cost and benefit terms is predicted by the model, which contrasts with the measured cost and benefit that show significant increases (Figure 1a). The observed discrepancies may indicate that cost and benefit exhibit a steeper dependence on operon expression than assumed in current models.

### Evolution in constant environments

**Figure 5:**
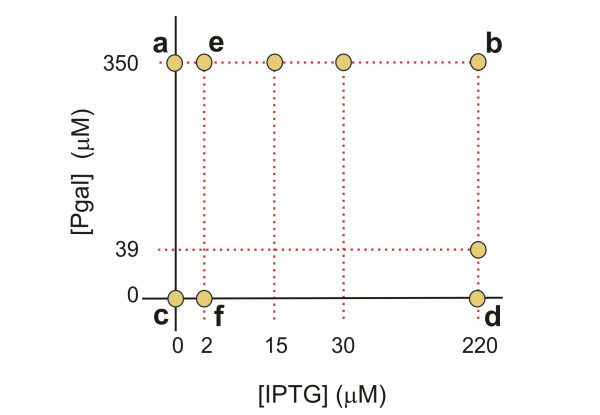
**Overview of the Pgal and IPTG concentrations in which adaptation experiments were performed**. Letters in the diagram refer to trajectories in Figure 6.

We performed serial dilution experiments in a number of constant environments with different concentrations of IPTG and Pgal, as indicated schematically in Figure 5. For each condition, a 10 ml culture was grown and diluted twice daily 300-500 fold for a total of ~800 generations. Every week a sample of each culture was stored at -80°C to preserve snapshots of its evolutionary history. The LacZ activity of the adapting populations was determined for different time points during the experiment.

**Figure 6:**
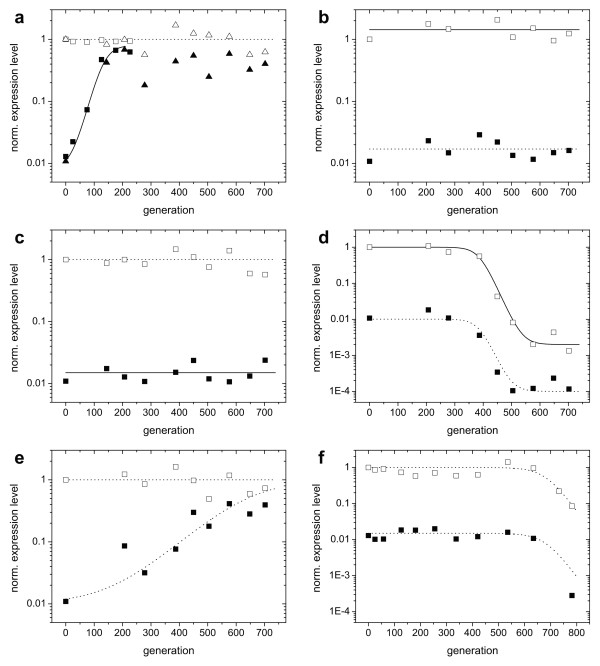
**Trajectories of expression levels (population averages) for populations adapting in constant environments**. Open symbols indicate the expression level induced with 220 μM IPTG, as assayed with a sample isolated from the evolving population. Solid symbols indicate the uninduced expression level, as assayed with a sample isolated from the population. Curves are fits based on growth rate differences under exponential growth (Additional file 1). Where induction levels are the same as in the environment to which the populations adapted, the curves are solid. Conditions during the evolutionary experiments: a) 0 μM IPTG, 350 μM Pgal. Two populations evolved in parallel are shown to yield the same adaptation dynamics (triangles and squares). b) 220 μM IPTG, 350 μM Pgal. c) 0 μM IPTG, 0 μM Pgal. d) 220 μM IPTG, 0 μM Pgal. e) 2 μM IPTG, 350 μM Pgal. f) 2 μM IPTG, 0 μM Pgal.

The induced and uninduced operon expression levels during the adaptation experiments are displayed in Figure 6. We first consider the environments with a high carbon source concentration (350 μM Pgal) and low induction (0 and 2 μM IPTG, Figure 6a and 6e). The uninduced expression for both experiments evolved to high levels that agree with predictions based on the optimality curve *Z*_opt_(*P*) (Figure 3). The induced expression state, which did not undergo selection, did not change and was maintained at the wild-type levels.

The two experiments (Figure 6a and 6e) showed differences in the evolutionary dynamics. The population grown without IPTG reached its optimal expression level in ~200 generations (Figure 6a). Notably, a second replicate experiment performed at this condition (squares and triangles in Figure 6a), is indistinguishable in its dynamics. The population grown at 2 μM IPTG (Figure 6e) reaches its final expression level only after more than 600 generations. If both traces are fitted with a simple competition model (assuming a single mutant fixation and a sufficiently high mutation rate to be able to neglect stochasticity due to bottlenecking the population, see Additional file 1), we find that the selection coefficient of the population growing without IPTG is more than 4 times larger than that of the population at 2 μM IPTG (*s *= 0.055 versus 0.013). Although one expects the selection coefficient to decrease for increasing concentrations of IPTG, the observed large difference between 0 and 2 μM IPTG is remarkable given the small wild-type expression differences for these IPTG concentrations (Figure 1b). However, Figure 1a shows that wild-type cells grown at a Pgal concentration of 0.5 mM already realize more than half of their expression benefit at 5 μM IPTG. Consequently, the additional selective advantage of abolishing repression is lowered correspondingly.

Figure 6b shows the evolutionary trace of a culture grown at a high carbon source concentration (350 μM Pgal) and high induction (220 μM IPTG). No significant adaptation was observed, which is consistent with the measured costs and benefits that predict near optimal growth rates for these conditions (Figure 2). When fully induced, the regulatory system is in principle free to lose regulation by neutral drift: mutations that deactivate the repressor do not affect the growth rate. Since mutations that restore repressor function are in general much less likely to occur, in the long run repressor null mutants may fix in the population. However, the expected rate at which this would occur is on the order of *1/μ *generations [21], where *μ *is the mutation rate towards *lacI*^-^mutants, which is ~1.10^-6 ^per cell per generation [22]. If repressor deactivation is neutral, fixation would therefore only be expected after 1.10^6 ^generations. Interestingly, a null mutation in the promoter controlling the transcription of the repressor may actually be selectively favored, since it should reduce the cost associated with the production of repressor protein. However, given the low amount of repressor protein (10-20 according to [23]) compared to the other *lac *gene products (induced LacZ expression is in the order of 1.10^4 ^per cell [24,25]), we expect that the selection coefficients associated with the loss of repressor production are too low to be observable within the time course of the experiments.

In the medium containing no IPTG and no Pgal, we find that the regulation remains unchanged (Figure 6c). This outcome is consistent with the predicted low selective pressures (Figure 2), as the expression of the *lac *operon products is tightly repressed under these conditions. We do find that expression is significantly reduced during growth on 200 μM IPTG and 0 μM Pgal (Figure 6d). Indeed, under these conditions the costs of this spurious operon expression are predicted to be significant, yielding a growth rate reduction of ~0.2 doublings per hour (Figure 1b and 2). The rate at which the expression decreases in the population suggests a selection coefficient of around 0.067. These values are comparable for the 0 μM IPTG and 350 μM Pgal medium which yielded an expression increase (Figure 6a) with an associated selection coefficient of ~0.055 and a predicted potential growth rate increase of ~0.2 doublings per hour (Figure 1b and 2). In contrast however, fixation of the decreased expression phenotype occurs at later generations, suggesting that it occurs less frequently than the increased expression phenotypes. This asymmetry might be understood by considering the mutational targets for obtaining increased and decreased expression. Increases in expression could be achieved by a diverse array of mutations in the repressor or the operator that lower the binding affinity, whereas decreases in expression would require more rare mutations that increase affinity or mutations in the *lacZ *promoter.

Figure 6f shows the evolutionary history of a population growing without Pgal, but with 2 μM IPTG. As in the case of 200 μM IPTG and no Pgal (Figure 6d), operon expression evolves to lower values. This observation is in line with the measured cost of spurious operon expression, which was shown to be significant even for these low inducer concentrations (Figure 1b). The measured costs are however lower than for 200 μM IPTG (Figure 6d), which predicts a lower selection coefficient. Indeed we observe a selection coefficient that is roughly half.

**Figure 7:**
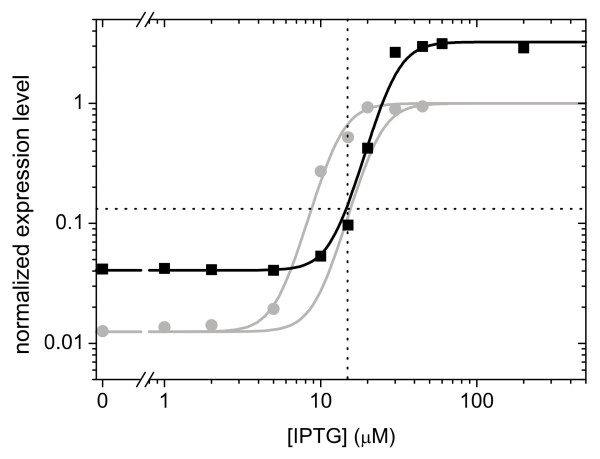
**Induction profile (black squares and curve) of a population evolved for approximately 700 generations in the presence of 15 μM IPTG and 350 μM Pgal**. Also shown is the wild-type induction profile without Pgal (grey data points and fit), as well as the predicted profile for wild-type incorporating Pgal anti-induction (grey curve shifted to the right). The expression levels at 15 μM IPTG for wild-type (with anti-induction) and evolved strain are very similar.

For the environment with 15 μM IPTG and 350 μM Pgal the evolved phenotype exhibited an altered induction profile (Figure 7). However, the profile changed in a way that the expression level at 15 μM IPTG in fact remained the same when taking into account Pgal anti-induction. From the growth data (Figure 2) we indeed expected a low selective pressure, as it indicates only a marginal difference in fitness between the expression level at 15 μM IPTG and the optimum that lies nearby at somewhat higher IPTG levels. Interestingly however, in this population a mutant had become fixed, thus suggesting a deviation from the predicted low level of selective pressure.

From eight clonal isolates after the serial dilution experiment we sequenced the chromosomal region consisting of the *lac *repressor, the *lac *promoter (upstream of *lacZ*), until 420 base pairs into the *lacZ *coding sequence. Compared to the reference GenBank nucleotide sequence of the *lac *operon (accession number J01636.1), all isolates contain a *lacI *polymorphism (C857T) that does not affect LacI function, and a synonymous mutation in the coding sequence of *lacZ*. From earlier work we know that C857T pre-existed in the MG1655 strain, and we assume that the synonymous *lacZ *mutation did also. Apart from these pre-existing mutations, three clones isolated from the population that adapted to 350 μM Pgal, 0 μM IPTG all showed a known hotspot frame shift deletion of four base pairs from a triply repeated TGGC (nucleotides 593-604 of the *lacI *coding sequence) [22]. This frame shift has been reported to lead to inactivation of the repressor [22], which is in line with our observation. One clone sequenced from adaptation on 350 μM Pgal, 220 μM IPTG and another from 0 μM Pgal, 0 μM IPTG, which retained wild-type induction characteristics, did not reveal any mutations. Remarkably, three clones sequenced from the population that adapted to 220 μM IPTG, 0 μM Pgal, also showed the hotspot frame shift. These isolates do not show a constitutive expression, but instead a greatly reduced expression, which means that they must carry another unidentified mutation. However, since these isolates did not contain mutations in the promoter controlling *lacZ *expression, no cause for the observed loss of LacZ activity (which originated from selection against expression cost, not against activity) can be identified at present.

### Optimality and evolution in alternating environments

**Figure 8:**
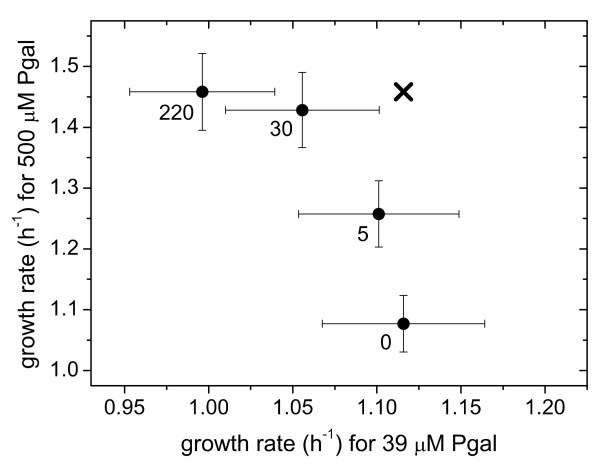
**Example of trade-offs experienced when expression is not regulated in an environment that alternates between a low Pgal concentration (39 μM) and a high Pgal concentration (500 μM)**. Data points denote the growth rate in each environment for a certain constant expression level (at the indicated concentrations of IPTG in μM). This trade-off data is directly obtained from Figure 1. Low expression levels (0 μM IPTG) yield optimal growth in medium with low Pgal concentrations, but non-optimal growth in medium containing high Pgal concentrations, and *vice versa *for high expression levels. Only when expression is regulated (low in low Pgal conditions and in high Pgal conditions), overall growth can be optimal over both environments, as is indicated by the black cross.

Variable environments may confront an organism with a trade-off: the possibility to improve in one environment, but at the expense of deteriorations in another. Here we can explicitly quantify such tradeoffs, which have been introduced conceptually by Levins [26], using the expression-growth relations (Figure 1b). We plotted the growth rate for a high Pgal concentration (500 μM) versus the growth rate in an environment with a low Pgal concentration (39 μM), for a range of IPTG levels (Figure 8). This graph indicates the growth rate combinations that are possible for phenotypes with one constant expression level in both environments (constituting a so-called Pareto-optimal front for the fitness in each environment), and can thus be used to determine the optimal unregulated phenotype. For instance, when the environment alternates between high and low Pgal for equal periods of time, this analysis predicts that the optimal constant expression level is achieved by inducing the *WT *system with 5 to 30 μM IPTG. Thus, at that expression level, the benefits minus the costs averaged over *two *environments are optimal. Importantly, the trade-off curve appears to have a concave shape, bulging out towards the cross in the upper right corner where growth in both environments is maximal. As a result, constant expression phenotypes can achieve near-maximal growth rates in each of the environments. This suggests that the superior responsive phenotype, which may achieve that maximum by regulating its expression to optimal values for both environments, has a limited selective advantage over optimal unregulated phenotypes. More generally, the analysis indicates that the selective advantages achieved by regulation are suppressed by the concave trade-off relation of this system.

**Figure 9:**
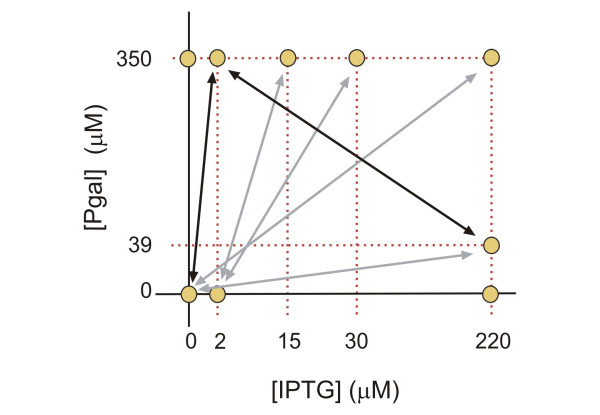
**Overview of Pgal and IPTG concentrations of the alternating environments in which adaptation experiments were performed**. Grey arrows indicate conditions which resulted in unaltered induction profiles. Black arrows did result in adapted profiles (see text).

We performed a number of serial dilution experiments in which the environment was alternating between two states (Figure 9). A change of environment was realized once or twice daily (see Materials and Methods). For four out of six experiments (marked with grey arrows in the Figure 9) we found no significant change of the induction profile. This evolutionary stasis can be explained using the measured expression-growth relation *g(I, P) *(Figure 1b and 2) and the results from the constant environments adaptation experiments. For example, at 2 μM IPTG and 0 μM Pgal there is a moderate selective pressure to decrease the low but spurious expression (Figure 1b and 2). On the other hand, at high Pgal (350 μM) with moderately high IPTG (15 and 30 μM), the induced expression is strongly favored to be maintained (Figure 1b and 2). This dominant selective force may explain why no overall decreases in expression were observed when alternating between these two environments. These conditions do produce a small selective advantage for phenotypes with an altered induction response that provide a decreased expression at 2 μM IPTG while maintaining the induced expression at 30 μM. Such adaptive change, however, was not observed. This may indicate limited genetic variation for such a phenotypic change, or reflect the limited benefit associated with such a change. A limited genetic variation is suggested by adaptation experiment in the 2 μM IPTG and no Pgal constant environment of Figure 6f, which shows a decreased expression phenotype emerges only at the end of the experiment (at the end of the 800 generations). This suggests that the genetic changes underlying expression decreases are rarer than for expression increases.

An additional rationale for the absence of evolutionary change might be found in bi-stability of the *lac *operon. Intermediate inducer concentrations have been shown to give rise to a bimodal phenotypic distribution for the *WT *genotype, in which the *lac *operon is either repressed or fully expressed [27]. In the media with 350 μM Pgal and 15 or 30 μM IPTG, a spontaneous fully expressed *WT *phenotype would have a high fitness and rapidly rise in number. Consequently, any fitness increase of an evolved regulatory mutant would be limited, and thus promote evolutionary stasis. However, it is unclear whether the growth conditions used here lead to bistability.

**Figure 10:**
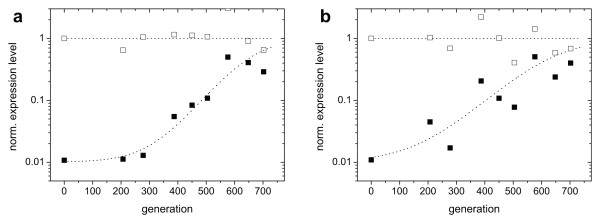
**Trajectories of expression levels for the populations evolved in alternating environments**. Open symbols represent induced expression levels (at 220 μM IPTG), solid symbols are uninduced expression levels. Curves are fits based on growth rate differences under exponential growth (Additional file 1). a) an environment alternating between 2 μM IPTG, 350 μM Pgal and 220 μM IPTG, 39 μM Pgal. b) an environment alternating between no IPTG, no Pgal and 2 μM IPTG, 350 μM Pgal. Both populations evolve towards a constitutive expression.

In two alternating environments the induction profile did change. First, alternating between 2 μM IPTG with 350 μM Pgal and 220 μM IPTG with 39 μM Pgal resulted in a high constitutive expression (Figure 10a). These conditions would in fact favor that expression increases without IPTG, and decreases with IPTG. The fact that only the former demand was met indicates a barrier to decreasing expression, which is consistent with results in constant environments. Moreover, one might expect less genetic variation for phenotypes that meet demands in two environments rather than one, which could explain the observed stasis in evolving the induction response. More prolonged adaptation experiments might clarify whether these constraints can be broken.

In the environment where no IPTG and no Pgal alternates with 2 μM IPTG and 350 μM Pgal, we also observe evolution towards a constitutive expression (Figure 10b). Here, the optimal regulated phenotype would have the inflection point of the induction curve shifted to lower IPTG concentrations, which could result from a higher affinity of the repressor for IPTG. The absence of these changes in our experiments despite the significant selective pressures, suggest that there is limited genetic variation for such phenotypes. The adaptation that occurred here maximizes growth in the environmental state with Pgal.

## Conclusions

In this study we first quantified how growth rates of *Escherichia coli *depend on the concentrations of an inducer that is not metabolized and a carbon source that does not induce. This decoupling allowed us to vary expression of the metabolizing enzyme at a chosen fixed carbon source concentration, and measure not only the cost of protein production but also the associated metabolic benefits directly. Without a decoupling between inducer and carbon source, measuring the benefits of incompletely induced expression is a challenge, as adding a carbon source has two effects: it not only leads to its metabolism, but also increases expression. Using this approach, we could directly determine the optimal expression level without relying on models for the dependence of costs or benefits on expression.

In the second part of this study, we tested whether evolution of the regulatory system can be understood in terms of the measured costs and benefits, by performing serial dilution assays in constant environments. We found that cells evolved in agreement with the cost-benefit analysis within the course of a few hundreds of generations. These results are in line with a recent study showing the *lac *operon expression evolves to the optimal level for a given concentration of lactose in the environment [4]. However, there are also important differences. In this earlier work, the *lac *operon was not regulated by the *lac *repressor, as full induction by IPTG resulted in constitutive expression. The underlying genetic changes occurred outside the regulatory region, in unspecified regions that apparently also provided control over *lac *operon expression. In contrast, here we investigated the adaptation of a *lac *operon whose expression was regulated by the *lac *repressors. We found that under these conditions adaptive genetic changes took place within the regulatory protein, specifically a hotspot frame shift deletion. This result is robust for varying degrees of initial suboptimality. These observations suggest that for transcriptionally regulated gene expression, genetic variation in the transcription factor provides a probable adaptive route.

The third part of this study involved the experimental evolution in temporally alternating environments, again using the serial dilution approach. We found adaptation to these conditions through changes in the overall expression level over both environments. We did not observe evolution of the response, i.e. independent expression changes in both environments. The results do provide insight into the causes underlying this evolutionary stasis. First, the experiments in constant environments revealed that while evolutionary expression increases could fix within 300 generations, expression decreases already required several hundred more generations, indicating that the underlying genetic changes are rarer. Altered responses likely involve even longer evolutionary periods, as one may expect that it requires even rarer genetic changes. Second, using the ability to vary the expression independently of the carbon source, we showed that the cross-environmental fitness tradeoff curve is concave. This suggests further increased evolutionary periods, as it indicates a limited selective advantage for phenotypes with altered responses over phenotypes that simply altered their overall expression. These findings thus provide a mechanistic rationale for the observed evolutionary stasis, and indicate which environmental conditions and system properties would be optimal for an experimental demonstration of the adaptive evolution of regulatory responses.

Analysis of the sustained genetic changes indicated a recurring hotspot frameshift mutation [22] (leading to *lacI^- ^*phenotypes) at a frequency that is an order of magnitude higher than would be expected from the genomic mutation rate and *lacI *target size (~10^-7 ^[28,29]), which is interesting in the light of regulatory evolution. In fact, both the deletion and addition of the 4 base pair repeat are observed at high frequency [22], which implies that reversals of hotspot mutations will also be more likely than reversing other *lacI *deactivations, as e.g. achieved by base pair substitutions. Together with the observation that the *lacI *coding sequence surrounding the hotspot is highly structured (palindromic), which is known to elevate the mutation frequency due to slippage of a replicating DNA polymerase [30], one may speculate that -in addition to regulation by sensing- the *lac *system also controls expression by what one could call 'mutational regulation', in which expression is switched stochastically and reversibly by genetic changes, in a manner that resembles the regulatory mechanisms underlying phase variation.

As a final remark, we note the possibility of a superior mutant that would undo the decoupling between inducer and carbon source and become responsive to Pgal, so that

(6) $$Z_{\text{reg}}\left(P\right)=Z_{\text{opt}}\left(P\right)$$

However, none of the evolved populations here was found to be induced by Pgal. Although this is an interesting possibility, we expect that for this type of mutation to occur, a range of environments and an amount of generations need to be surveyed that is not easily accessible in this type of laboratory evolution experiments. Adaptation of inducer specificity may be more effectively studied at a higher level of control over the system, for example using targeted mutagenesis.

Here we presented a method to measure the optimal expression level of a vital operon, and to design environments with a well-defined performance mismatch, which is based on the decoupling between the sensed cue and the metabolized carbon source. This principle is general, and may be applied to study many open issues regarding the evolution in complex environments in a more quantitative manner.

## Methods

### Strains and media

We used *Escherichia coli *strain MG1655 [19]. All experiments were performed in M9 minimal medium, consisting of M9 salts (Sigma-Aldrich), supplemented with 0.1 mM CaCl_2 _(Merck Eurolab), 1 mM MgSO_4 _(Merck Eurolab), and 5 g/l casamino acids (BD Biosciences). When indicated, media contained isopropyl-β-D-thiogalactopyranoside (IPTG) and phenyl-β-D-galactoside (Pgal), both obtained from Sigma-Aldrich. All cultures were grown at 37°C. As an initial fast adaptation to the medium was observed, evolutionary runs were started after growth for ~30 generations on minimal medium without Pgal or IPTG.

### Determination of growth rates

Growth rate determinations were performed after overnight growth in the medium described above, without IPTG or Pgal, followed by at least 3 hours growth in medium with the appropriate concentrations of Pgal and IPTG. Subsequently the cultures were diluted to an optical density of ~5.10^-4 ^and transferred to a pre-warmed flat bottom 96 well microtiter plate (VWR 351172), at 200 μl per well. Optical density at 600 nm was recorded in a Perkin & Elmer Victor^3 ^plate reader every 4 minutes, and every 29 minutes 9 μl sterile water was added to each well to counteract evaporation. When not measuring, the plate reader was shaking the plate at double orbit with a diameter of 2 mm. All presented growth values are averages of three independent measurements. From measurements in which all 96 wells were inoculated with wild-type MG1655, we determined the error margins on our averaged growth data to be 4.3%.

### Determination of beta-galactosidase activity

The β-galactosidase activity of mutant pools and clones was determined using the fluorogenic substrate fluorescein-di-β-D-galactopyranoside (FDG, MarkerGene Technologies Inc, Eugene, OR, USA), which allows for an accurate determination of the LacZ activity over at least 4.5 orders of magnitude. Before transfer to a 96 well plate, cultures were grown overnight without IPTG and Pgal, and then diluted to an optical density of ~5.10^-4^. When expression levels were high so that overnight passage through stationary phase resulted in 'superinduced' LacZ activity levels (see Additional file 1), growth times before fluorescence determination were prolonged. Cells were fixed by addition of formaldehyde to 0.15%. LacZ activity is proportional to the maximum slope of FDG-fluorescence curves. Further details are given in Additional file 1.

### Serial dilution experiments

10 ml cultures were grown in 50 ml polypropylene tubes in a 37°C water bath under vigorous shaking (200 rpm). Cultures were diluted 300-500× twice daily in fresh medium. As stationary cultures contain ~10^9 ^cells ml^-1^, this implies bottleneck sizes of ~10^7 ^cells (for 10 ml total culture volume). The alternating conditions were either switched twice daily (for the cultures alternating between 2 μM IPTG, 0 μM Pgal and 15/30 μM IPTG, 350 μM Pgal, see Figure 9), or once daily (for the remaining conditions). When switching from a higher concentration of IPTG or Pgal to a lower one, cultures were washed three times in minimal medium. Every four days a sample of the cultures was frozen at -80°C. Re-inoculation occurred after thawing and 3× washing in minimal medium.

## Authors' contributions

FJP, DJK, and SJT conceived and designed the research, PDH and MGJV performed the experiments, FJP, PDH, MGJV, and SJT analyzed the data, FJP, MGJV, and SJT wrote the paper. All authors read and approved the final manuscript.

## Supplementary Material

Additional file 1 **Supplementary Information and Figures**. Supplementary Information Supplementary Figure S1 Supplementary Figure S2 Supplementary Figure S3. Click here for file
