# Supplementary material for: Optimality and evolution of transcriptionally regulated gene expression
Source: BMC Syst Biol. 2011 Aug 16;5:128. doi: 10.1186/1752-0509-5-128 (PMC3182923; doi:10.1186/1752-0509-5-128)
Supplement: Additional file 1 — Supplementary Information and Figures. Supplementary Information Supplementary Figure S1 Supplementary Figure S2 Supplementary Figure S3. [file 1752-0509-5-128-S1.DOC]

Optimality and evolution

of transcriptionally regulated gene expression

Frank J. Poelwijk, Philip D. Heyning, Marjon G.J. de Vos,

Daniel J. Kiviet, and Sander J. Tans***

AMOLF Institute

Science Park 104, 1098 SG, Amsterdam, The Netherlands

***To whom correspondence should be addressed; E-mail: tans@amolf.nl.

# Current address: Green Center for Systems Biology and Department of Pharmacology, University of Texas Southwestern Medical Center, Dallas, TX 75390-9050, USA

**Supplementary Material**

**Analysis of induction curves and competitive inhibition by Pgal**

The *lac* repressor does not only bind IPTG, it also has a affinity for Pgal. Pgal does not induce the repressor, but does competitively prevent IPTG from binding and thus effectively anti-induces the repressor. Since the equilibrium dissociation constants differ by three orders of magnitude ($K_{D}$ = 110 for IPTG and 110 for Pgal (Miller 1978)), this effect is only noticeable when the Pgal concentration is much higher than the IPTG concentration. In other cases IPTG and Pgal can be considered to be decoupled with respect to induction of the *lac* repressor. We measured the effect of Pgal anti-induction by growing cultures under different concentrations of Pgal and IPTG. Immediately before the LacZ assay, all concentrations of IPTG and Pgal were equalized to prevent unequal inhibition at the level of LacZ. The results are given in Figure S1.

The data for Pgal = 0 was fitted with a general Hill function (Kuhlman 2007)

$\alpha_{\text{IPTG}}=\alpha_{0} \frac{1+F ([\text{IPTG}]/C_{\text{IPTG}})^{m}}{1+([\text{IPTG}]/C_{\text{IPTG}})^{m}}$ (S1)

where $\alpha_{0}$ is a parameter relating the number of LacZ molecules to the measured LacZ activity, *F* is the ratio between induced and uninduced LacZ activity, $C_{\text{IPTG}}$ is a dissociation constant associated with the affinity of IPTG to the repressor, and $m$ is a phenomenological Hill exponent incorporating non-linear behavior of the *lac* induction (due to e.g. cooperative binding of the repressor to multiple operators).

The curves for higher Pgal concentrations in Figure S1 were obtained by incorporating Pgal anti-induction into the Hill description of the system. Pgal anti-induction increases the effective equilibrium dissociation constant of IPTG to the *lac* repressor (or equivalently lowers the effective IPTG concentration).

$C_{\text{IPTG,eff}}=C_{\text{IPTG}}(1+\frac{[\text{Pgal}]}{K_{P}})$ (S2)

where $K_{P}$ is the apparent equilibrium dissociation constant of Pgal binding to the repressor (which incorporates a potential difference in internal and external Pgal concentrations).

All curves in Figure S1 could be fitted using a $K_{P}$ of 0.45 mM. A minor vertical offset of the curves was observed due to the different growth rates under the different (Pgal, IPTG) conditions, as can be expected on the basis of a slightly different dilution rate associated with cell division.

**Comparison to reaction kinetics model for transport and degradation**

Here we modeled our system according to the cost-benefit analysis for a fixed expression level as reported in (Dekel 2005). We modify this model by including induction by IPTG and anti-induction by Pgal. A comparison of this model to the obtained growth data in Figure 1 is made.

The proposed (Dekel 2005) functional form for relative growth due to the cost and benefit of *lac* operon gene expression is

$\Delta g=-\eta(Z)+B(Z,L)=-\frac{\eta_{0}\cdot Z}{1-Z/M}+\delta\frac{Z\cdot L}{K_{Y}+L}$ (S3)

where $\eta$ is the cost term and $B$ the benefit term, that depend on the concentration of LacZ ($Z$), lactose ($L$), and the equilibrium dissociation constant of the LacY permease and lactose ($K_{Y}$). This expression was derived under the assumption of low lactose concentrations ($<$ 1 mM). This assumption assures that the rate limiting step in lactose metabolism is the import of lactose into the cell by LacY. In our system this condition is also fulfilled, as the equilibrium dissociation constant of LacY for Pgal is of the same order of magnitude as that for lactose, being 1.3 mM (Sahin-Tóth 2002), while the dissociation and rate constants of LacZ for lactose and Pgal are comparable (Tenu 1971, Martinez-Bilbao 1991). Hence the assumption for Pgal is justified.

We thus modified the expression for relative growth as follows

$\Delta g=-\eta(Z(I,P))+B(Z(I,P),P)=-\frac{\eta_{0}\cdot Z(I,P)}{1-Z(I,P)/M}+\delta\frac{Z(I,P))\cdot P}{K_{Y}+P}$ (S4)

where the LacZ expression $Z(I,P)$ depends on IPTG as well as on Pgal concentration (the latter being of influence only at high concentrations). Using this model we fitted the growth at high IPTG concentrations (220 μM ), which works well for Pgal concentrations up until 1 mM (Figure 4). Indeed for higher concentrations the assumptions of the model may be violated.

When we compare model predictions using the obtained parameters from the fit for 200 μM IPTG to reproduce the data at lower IPTG concentrations, we observe a qualitative correspondence only (Figure S2). The major difference is the occurrence of a cost and a benefit at very low IPTG concentrations (e.g. 5 μM), while LacZ expression levels have only increased marginally (from Figure S1 we see that they are still a factor ~100 below fully induced levels). These observations suggest that the cost and benefit terms may exhibit a steeper dependence on operon expression levels than assumed in the model. Alternatively, the model might need to incorporate competition between Pgal and IPTG for LacZ. However this would imply that Pgal import by LacY is not rate limiting, and hence violate the assumptions underlying the present model, which precludes an analytical solution. A more complete description of the system is beyond the scope of this work.

**Non-stochastic competition model**

Evolutionary traces were fitted (dotted and solid curves in expression history graphs) using a non-stochastic model for the change in expression when a mutant fixes in the population. It is known that the fate of mutants in a population that is periodically bottlenecked is influenced by 'sampling noise' when the mutation is initially only present in a few individuals (Azevedo 2002). However, when the mutation rate is such that the expected number of mutants after bottlenecking is significantly larger than 1 (*μb* >> 1 where $b$ is the bottleneck size and $\mu$ the mutation rate), these stochastic effects can be ignored. This seems to hold in our case at least for the hotspot mutations that occur at a rate of ~110, while our bottleneck size is ~10. Moreover, the selection coefficients are estimated from the rate of the fixation process, which is independent of the mutation rate (unless the population is very small or the mutation rate very high).

As both the wild-type and the mutant population grow exponentially in between the bottlenecks we have for their numbers

$$\text{wild}-\text{type} N(t)=N_{0}\text{e}^{\text{ln}2gt}$$

$\text{mutant} N^{*}(t)=N_{0}^{*}\text{e}^{\text{ln}2g(1+s)t}$ (S5)

where $g$ is the wild-type growth rate, $s$ is the selection coefficient. On the basis of these numbers of individuals, we have for the expression levels of a population average

$E_{\text{ave}}(t)=\frac{E^{*}N^{*}(t)+EN(t)}{N^{*}(t)+N(t)}=E \frac{E_{r}R_{0}\text{e}^{\text{ln}2gst}+1}{R_{0}\text{e}^{\text{ln}2gst}+1}$ (S6)

where $R_{0}=N_{0}^{*}/N_{0}$ is the initial ratio of mutants, and $E_{r}=E^{*}/E$ the ratio of the expression levels of the mutant and the wild-type. This expression assumes that a mutant arises close to the start of the experiment and further does not address consecutive mutations.

**Enzyme dilution**

Important for both the correct determination of expression levels, as well as important to take into account when setting up an experiment with alternating medium conditions, is the fact that we observed a 'superinduced' LacZ activity for cells after spending a stationary phase at high expression levels. We found that the expression levels of induced cells as determined immediately after they leave stationary phase, can be up to a factor of 10 higher that the expression during exponential growth. If this happens, it can take very long before LacZ molecules are diluted out by cellular division, even when their production is low. To demonstrate this effect, a culture of wild-type cells was grown overnight at full induction (200 μM IPTG). The next morning the culture was washed and grown in fresh medium without IPTG. At specific time points samples were taken and frozen at -80C. Afterwards the expression levels for these samples were determined (Figure S3). In the figure induced and uninduced levels of expression for an exponentially growing population are given as dotted lines. We indeed observe that the cells initially have a much higher LacZ expression than exponentially growing induced cells. The expression levels decrease over time, which corresponds to the observed growth rate of the cells. Remarkably, even after 8 hours of growth the expression level of exponentially growing uninduced cells has not yet been reached.

In our determination of expression levels (see above), we have taken into account the long times it may take to be able to determine the expression levels associated with exponential growth. Importantly, for evolution experiments under alternating conditions, it is essential to take into account enzyme dilution effects on the response times of the regulatory system.

**Determination of** β**-galactosidase activity**

To determine the β-galactosidase activity (and thus the expression level) of mutant pools and clones in our experiments, we used the fluorogenic substrate fluorescein-di-β-D-galactopyranoside (FDG), which allows for an accurate determination of the LacZ activity over at least 4.5 orders of magnitude. FDG contains two galactose groups that both have to be cleaved in order to release the fluorescein.

$$\text{FDG}+E\to\text{FMG}+E+\text{galactose}$$

$$\text{FMG}+E\to\text{fluorescein}+E+\text{galactose}$$

An extended model for the FDG-FMG hydrolysis has previously been proposed (Huang 1991). In our concentration range of LacZ and FDG, the increase in fluorescence is given by (eq. 7 in ref. (Huang 1991)):

$\frac{d}{dt}F=k_{2}E\frac{S_{0}}{K_{m}+S_{0}}(\alpha_{P}+(\alpha_{M}-\alpha_{P})e^{-Rt})$ (S7)

where $R$ is the relaxation constant (time scale to reach maximum fluorescence rate), $E$ is the (total) concentration of enzyme, $k_{2}$ is the catalysis rate constant of FDG to FMG, and the $\alpha$'s are proportionality factors between product and fluorescence, in the paper given as $F=\alpha_{P}P+\alpha_{M}M$ ($P$ is product (fluorescein) and M is FMG). $K_{m}$ is the Michaelis-Menten constant for FDG and $S_{0}$ is the initial FDG concentration. We can see that at time $t$=$0$ as well as at large $t$'s the rate is proportional to $E$, though with different proportionality constants (first $\alpha_{M}$, then $\alpha_{P}$).

Ref. (Huang 1991) gives measured values for $\alpha_{M}$= 5.3 μM and $\alpha_{P}$= 150 μM. Although assigning arbitrary units to the fluorescence counts, they are relevant as relative quantities between FMG and fluorescein. Thus at $t$=$\infty$, equation (S7) reduces to

$\frac{d}{dt}F=\alpha_{P} k_{2}E\frac{S_{0}}{K_{m}+S_{0}}$ (S8)

In order to determine the enzyme concentration per cell, fitted slopes are divided by the cell density. We use here $\varepsilon\propto E/\text{OD}_{600}$, where $\varepsilon$ is the LacZ concentration per cell.

Immediately prior to adding the fluorogenic substrate, we added IPTG and Pgal so that in each sample their concentrations are equal, to prevent unfair comparison due to competitive inhibition of LacZ by IPTG or Pgal.

**Supplemental Figures**


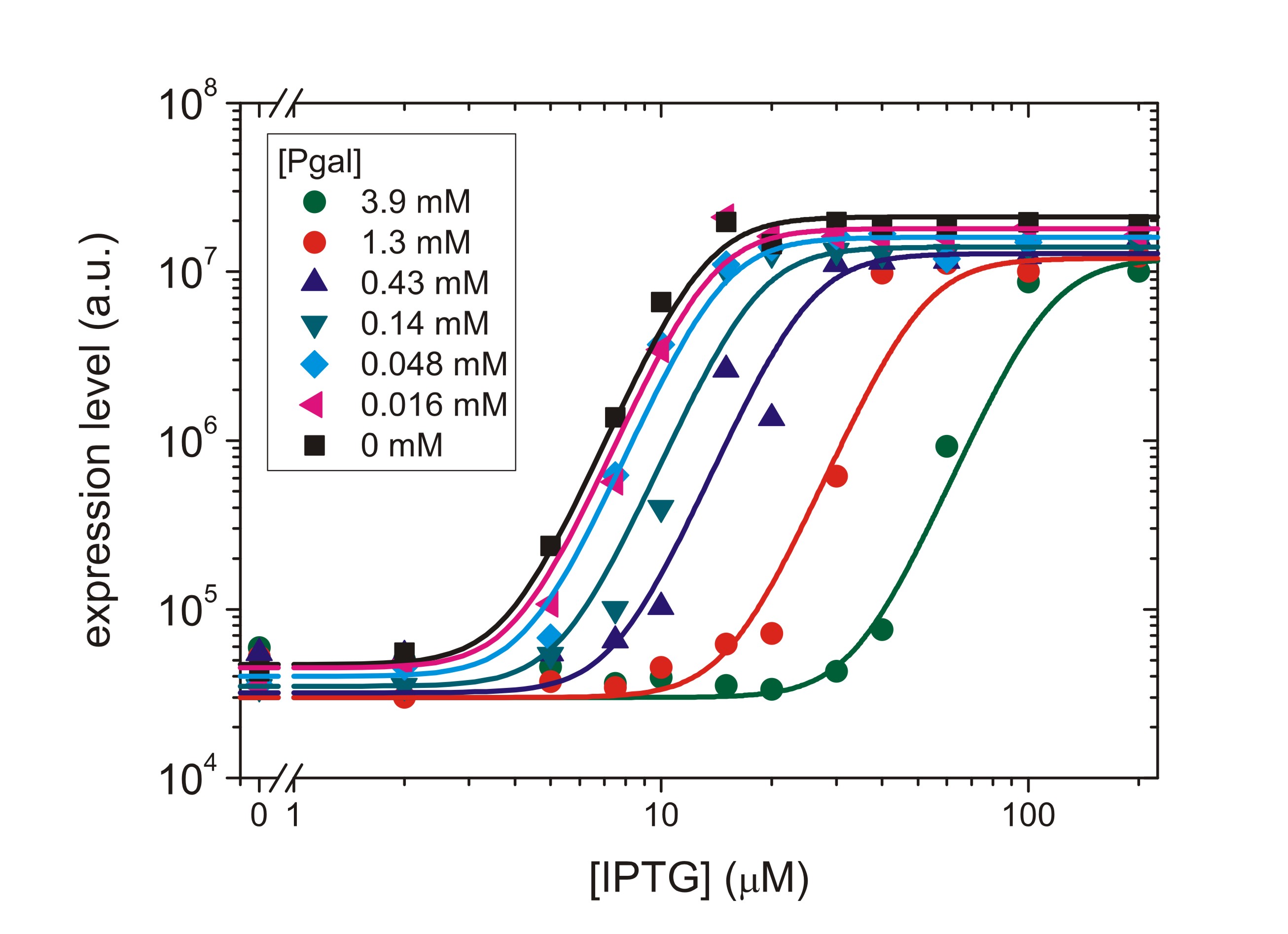


Figure S1. Induction profiles for wild-type cultures grown at different Pgal concentrations. The data is fitted with a Hill function that incorporates anti-induction by Pgal.


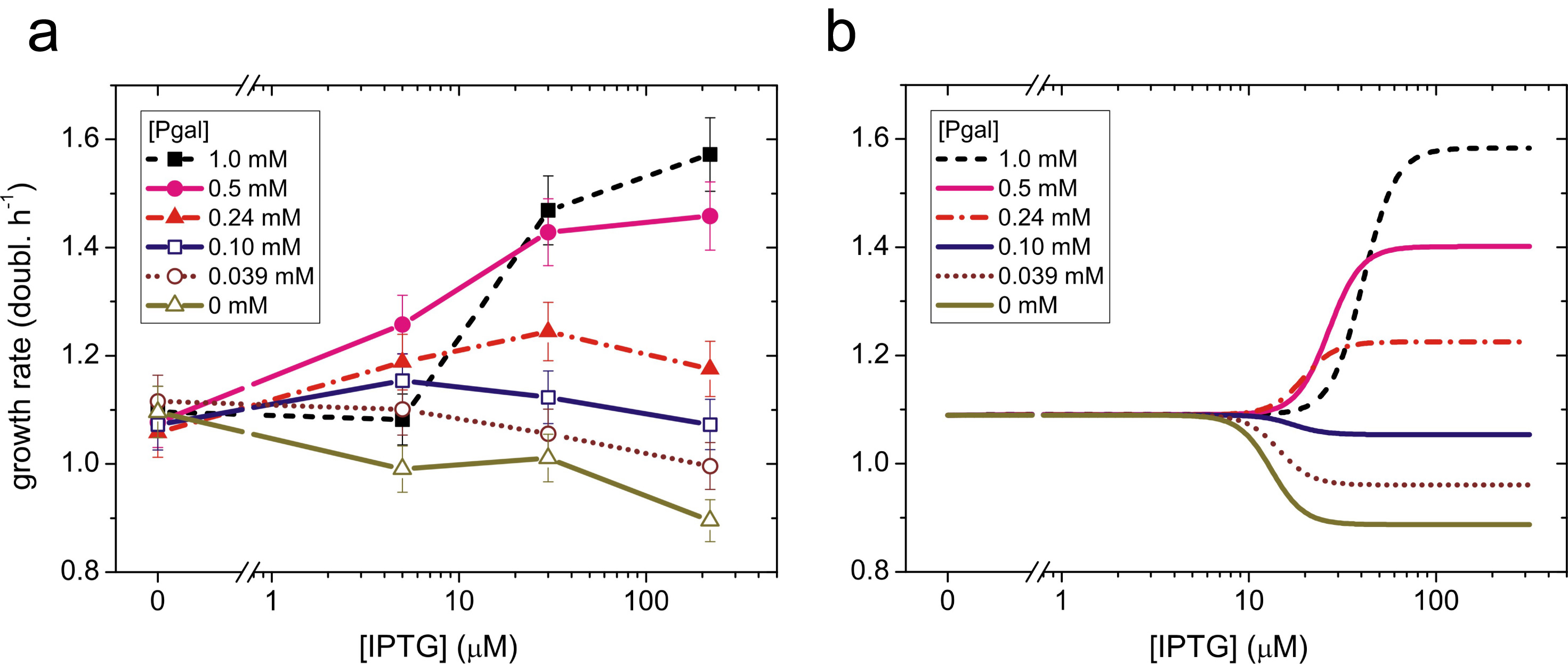


Figure S2. Comparison of growth data in the presence of various concentrations of IPTG and Pgal (left), with a model incorporating transport and catalysis of Pgal, as well as induction by IPTG and anti-induction by Pgal (right). The qualitative trend and the predictions at higher IPTG concentrations correspond well. For lower IPTG concentration there is a discrepancy: cost and benefit of expression occur at lower induction levels than is predicted by the model, on the basis of the measured induction profiles (Figure S1).


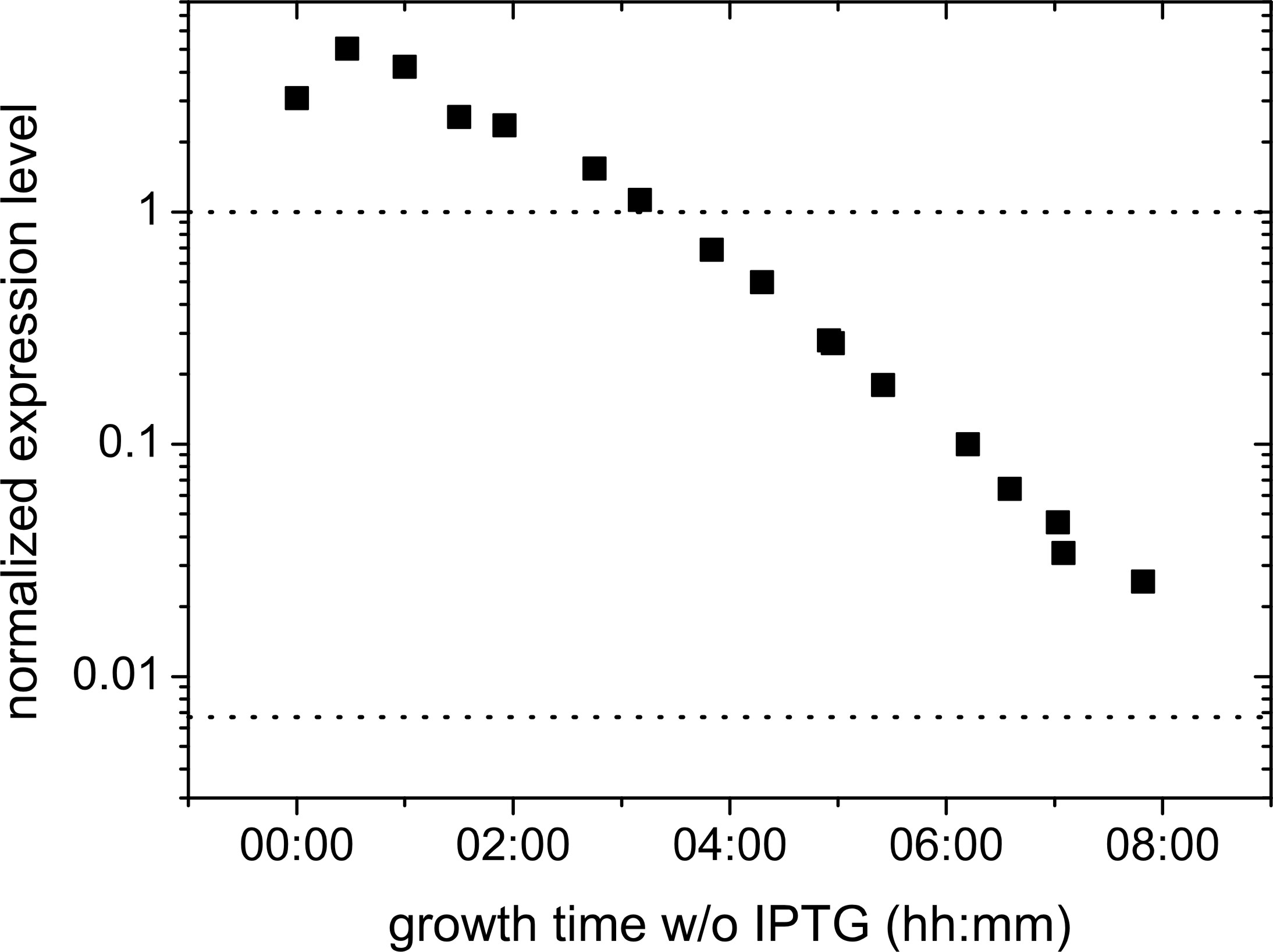


Figure S3. Enzyme dilution by cellular division, visualized by decreasing expression levels. At t=0 a stationary overnight culture of induced (200 μM) wild-type cells is inoculated in fresh medium. At the indicated time points, samples are taken from this culture of which the expression is determined. Dotted lines are uninduced (lower) and fully induced (upper) expression levels of an exponentially growing wild-type population. On the logarithmic vertical scale we observe a near linear decay, corresponding to exponential dilution of the enzyme. From the decay rates a slowly increasing decay rate (1/t) was determined, starting at 0.92 h and ending at 1.3 h . The initial rate corresponds well to the growth rate of the population at full induction, whereas the end rate is somewhat higher than that of an uninduced population, which might be caused by intrinsic degradation of LacZ.

**References**

Azevedo, R. B. R., Keightley, P. D., Wahl, L. M., Gerrish, P. J. and Saika-Voivod, I. 2002. Evaluating the impact of population bottlenecks in experimental evolution. Genetics 162: 961-971.

Dekel, E. and Alon, U. 2005. Optimality and evolutionary tuning of the expression level of a protein. Nature 436(7050): 588-592.

Huang, Z. J. 1991. Kinetic fluorescence measurement of fluorescein di-beta-D-galactoside hydrolysis by beta-galactosidase: intermediate channeling in stepwise catalysis by a free single enzyme. Biochemistry 30: 8535-8540.

Kuhlman, T., Zhang, Z., Saier, M. H. and Hwa, T. 2007. Combinatorial transcriptional control of the lactose operon of *Escherichia coli*. Proc. Natl. Acad. Sci. U.S.A. 104: 6043-6048.

Martinez-Bilbao, M., Holdsworth, R.E., Edwards, L.A. and Huber, R.E. 1991. A highly reactive β-galactosidase (*Escherichia coli*) resulting from a substitution of an aspartic acid for Gly-794. J. Biol. Chem. 266: 4979-4986.

Miller, J. H. and Reznikoff, W. S. 1978. The Operon. New York, Cold Spring Harbor Laboratory Press.

Sahin-Tóth, M., Gunawan, P., Lawrence, M. C., Toyokuni, T. and Kaback, H. R. 2002. Binding of hydrophobic D-galactopyranosides to the lactose permease of *Escherichia coli*. Biochemistry 41(43): 13039-13045.

Tenu, J. P., Viratelle, O. M., Garnier, J. and Yon, J. 1971. pH dependence of the activity of beta-galactosidase from *Escherichia coli*. Eur. J. Biochem. 20: 363-370.
